# Adefovir dipivoxil-associated Fanconi syndrome combined with peripheral neuropathy: a case report and literature review

**DOI:** 10.3389/fphar.2025.1585126

**Published:** 2025-08-01

**Authors:** Shanshan Fu, Guanao Wu, Yuanqi Zhao, Min Zhao

**Affiliations:** ^1^ The Second Clinical College of Guangzhou University of Chinese Medicine, Guangzhou, China; ^2^ Department of Neurology, The Second Affiliated Hospital of Guangzhou University of Chinese Medicine, Guangdong Provincial Hospital of Chinese Medicine, Guangzhou, China

**Keywords:** Fanconi syndrome, adefovir dipivoxil, peripheral neuropathy, hypophosphatemia, energy metabolism

## Abstract

Long-term use of low-dose adefovir dipivoxil (ADV) has been increasingly associated with Fanconi syndrome; however, cases of Fanconi syndrome combined with peripheral neuropathy remain rare. Here, we report a 67-year-old man who developed progressive limb weakness and sensory abnormalities after approximately 6 years of treatment with 10 mg ADV. He was diagnosed with ADV-associated Fanconi syndrome accompanied by peripheral neuropathy. Following the substitution of ADV with entecavir and 5 months to 6 months of phosphate supplementation, the patient’s muscle weakness nearly resolved, and peripheral nerve damage showed significant improvement.

## 1 Introduction

Fanconi syndrome is a proximal tubule disorder characterized by excessive excretion of bicarbonate, phosphate, glucose, uric acid, urinary potassium, urinary sodium, and some amino acids from the urine (Foreman, 2019). ADV, a nucleotide analog used to treat wild-type and lamivudine-resistant chronic Hepatitis B Virus (HBV) infection (Barcena et al., 2005), can induce acquired Fanconi syndrome by impairing renal tubular reabsorption. Reports of ADV-related Fanconi syndrome have increased in recent years. A retrospective study found that 80.9% of patients initially presented with bone pain, typically affecting the hips, knees, ankles, and ribs, due to disrupted bone metabolism (Sun et al., 2020). Notably, some studies have reported neurological abnormalities in these patients, including muscle weakness, sensory changes, abnormal reflexes, and pathological signs, suggesting a potential link between Fanconi syndrome and neurological disorders (Chen N. et al., 2018).

In our department, we admitted a patient with ADV-related Fanconi syndrome combined with peripheral neuropathy, initially misdiagnosed with lumbar disc herniation and osteoporosis due to lower extremity weakness. This case highlights the importance of recognizing such associations and provides valuable diagnostic and therapeutic insights.

## 2 Case report

**FIGURE 1:**
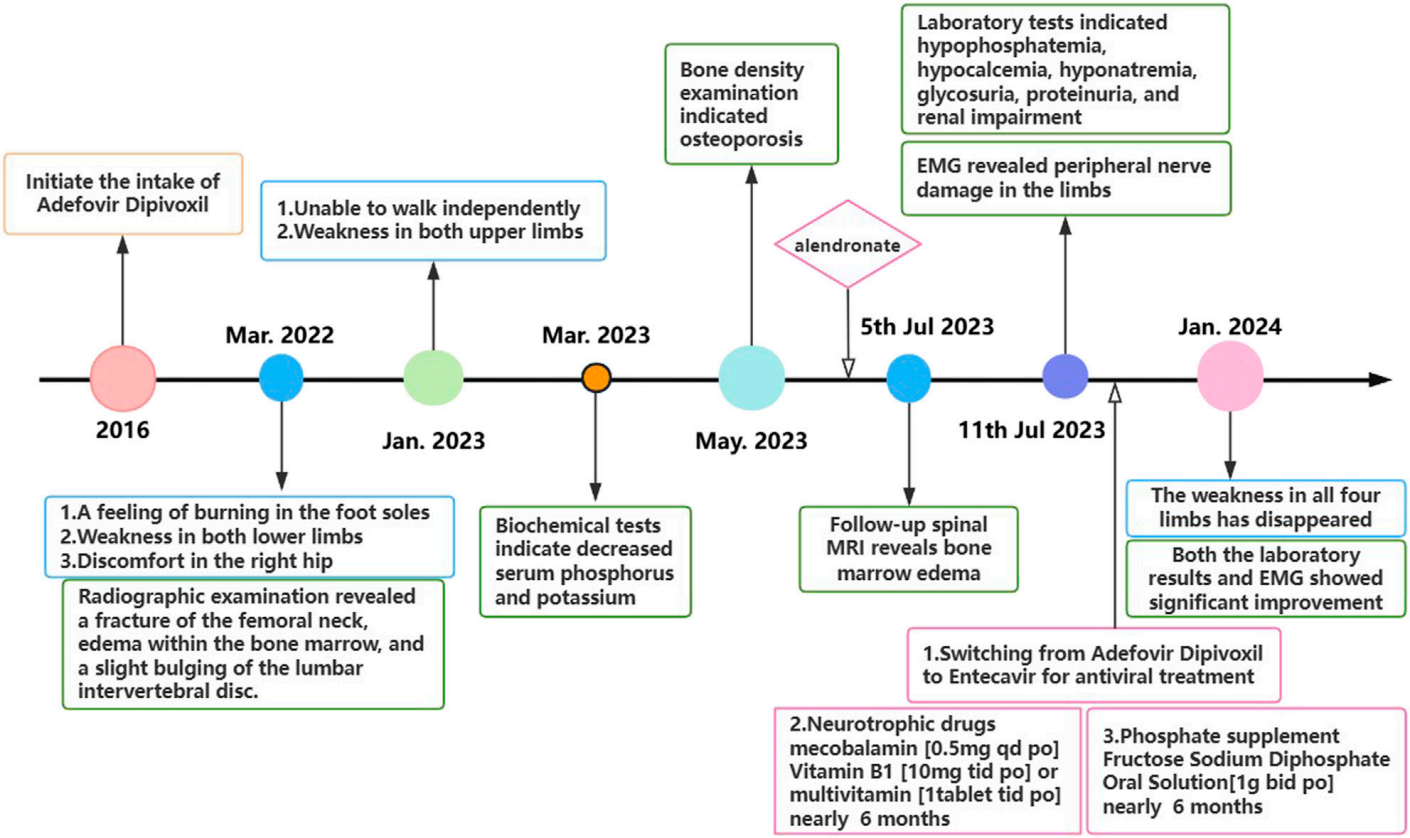
The patient’s diagnostic and therapeutic process.

In July 2022, a 67-year-old man presented with progressive bilateral lower extremity weakness, accompanied by coldness and heaviness in the distal lower limbs, right hip discomfort, and bilateral plantar burning sensation. Imaging studies at a local hospital revealed lumbar disc bulging, a right femoral neck fracture, and bone marrow edema in the lumbar vertebra and femoral head. Despite symptomatic treatment, his condition still worsened. In January 2023, he was unable to walk independently and began to experience weakness in both upper limbs, although fine motor skills such as buttoning remained intact. Between March and July 2023, the man with persistent limb weakness was found to have hypophosphatemia, hypokalemia, and osteoporosis (Bone mineral density of the right femoral head was measured at 0.571 g/cm^2^, with a T-score of −3.2.), but the aforementioned clinical symptoms persisted after corresponding drug therapy (Figure 1). On 11 July 2023, he visited the orthopedic outpatient clinic of our hospital and received the electromyography (EMG) that demonstrated multiple peripheral damages to the nerves of the limbs (Table 1). Therefore he was admitted to our department with a primary diagnosis of “Unexplained Muscle Weakness” and received a physical examination. Physical examination showed reduced muscle bulk in all limbs, neck flexors and right proximal upper limb strength grade 3, bilateral lower limb strength grade 4, brisk deep tendon reflexes in the upper limbs and hyperactive reflexes in the lower limbs. No other abnormalities noted. The patient had a 6-year history of hepatitis B and had been taking 10 mg ADV daily for nearly 6 years. He denied other medical or family histories of hereditary diseases.

**TABLE 1:** Electromyography data.

Program	Examination time	
	Jul. 2023 (left/right)	Jan. 2024 (left/right)
Median nerve		
CMAP (mV, wrist)	5.8/4.4	7.1/6.4
CMAP (mV, elbow)	ND/4.3	6.7/6.2
DML (ms, wrist to elbow)	ND/9.3	4.6/4.4
MCV (m/s, wrist to elbow)	ND/53	**48/48**
SCV (m/s, little finger to wrist)	52**/42**	52/**43**
SNAP (μV, finger to wrist)	23/**10**	30.5/**8.7**
F - Latency (ms)	33.2/29.3	30.1/27.3
F%	**75**/93.8	100/100
Ulnar Nerve		
CMAP (mV, wrist)	10.5/9.4	12.7/10.9
CMAP (mV, below elbow)	8.6/8.1	9.1/8.1
CMAP (mV, above elbow)	9.3/8.2	8.5/7.7
DML (ms, wrist)	7.7/7.7	4.1/3.9
MCV (m/s, wrist to below elbow)	48/49	51/50
MCV (m/s, wrist to above elbow)	48/53	50/53
SCV (m/s, little finger to wrist)	**NR/NR**	**33/30**
SNAP (μV, finger to wrist)	**NR/NR**	**16.1/10.1**
F-Latency (ms)	30.5/30.5	30.5/28.9
F%	100/87.5	91.7/100
Peroneal Nerve		
CMAP (mV)	ND/**2.2**	2.3/**1.7**
DML (ms)	ND/7.8	9.0/8.5
MCV (m/s)	ND/**33**	**32/34**
SCV (m/s)	**NR/NR**	44/45
SNAP (μV)	**NR/NR**	12.5/10.5
Sural Nerve		
SCV (m/s)	**39/40**	47/50
SNAP (μV)	12/**5**	13.2/16.9
Tibial Nerve		
CMAP (mV)	3.4/3.7	3.9/3.3
DML (ms)	8.4/10.4	9.9/10.7
MCV (m/s)	**34/36**	36/35

CMAP, compound motor active potential; DML, distal motor latency; MCV, motor nerve conduction velocity; SCV, sensory nerve conduction velocity; SNAP, sensory nerve active potential. ND, not examined; NR, not elicited.

Notes: Bold values in the table indicate abnormal results.

**FIGURE 2:**
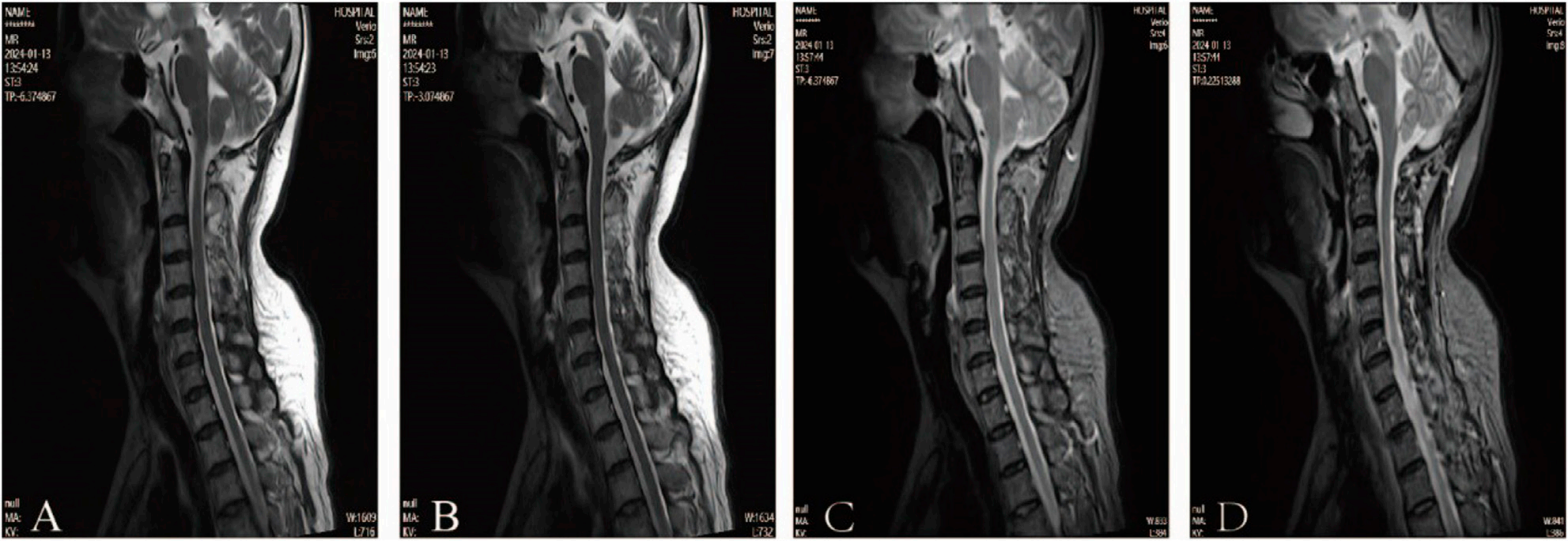
Magnetic resonance imagings of the cervical cord in July, 2023 **(A–D)**.

According to clinical evidence collected, the lesion was preliminarily localized to the spinal cord and peripheral nerves. The patient’s cerebrospinal fluid (CSF) analysis revealed normal glucose, chloride, and white blood cell count, but elevated protein (811 mg/L; normal 150–450). Oligoclonal bands (CSF-restricted) and elevated IgG (93.3 mg/L; normal 10–30) were observed. Spinal MRI revealed no significant abnormalities, ruling out spinal cord lesions (Figure 2). The EMG revealed multiple peripheral damages with preserved F-wave responses in all tested nerves except for the median nerve which showed decreased F-wave persistence (Table 1). Peripheral neuropathy was confirmed, but the etiology still remains undetermined after excluding metabolic, vascular, infectious, and immune-related factors (e.g., serum ganglioside antibodies, node of Ranvier antibodies and other autoimmune antibodies).

During the examination, we incidentally identified metabolic acidosis, hypophosphatemia, hypocalcemia, hyponatremia, glycosuria, proteinuria, and renal impairment (Table 2). Given his 6-year history of ADV therapy, ADV-associated Fanconi syndrome was suspected. Consequently, we replaced ADV with entecavir, administered sodium fructose diphosphate oral solution [1 g bid po] for phosphorus supplementation and other symptomatic treatments. After 6 months, the patient’s limb weakness was nearly resolved, and both laboratory results and EMG findings showed significant improvement compared to previous assessments.

**TABLE 2:** Laboratory data.

	Jul. 2023	Jan. 2024	References range
Serum			
pH	**7.327**	—	7.350–7.450
Potassium (mmol/L)	3.61	3.67	3.5–5.3
Sodium (mmol/L)	141	142	137–147
Chloride (mmol/L)	**114.2**	108.3	99–110
Phosphate (mmol/L)	**0.24**	0.94	0.85–1.51
Calcium (mmol/L)	**1.9**	2.28	2.11–2.52
magnesium (mmol/L)	0.94	0.92	0.75–1.02
25-hydroxyvitamin D (mmol/L)	**69**	81.1	≥75
alkaline phosphatase (U/L)	122	**166**	45–125
parathyroid hormone (pg/mL)	73.3	28.0	18.5–88.0
Uric Acid (mmol/L)	**95**	**163**	208–428
creatinine (μmol/L)	**145**	**116**	57–111
estimated glomerular filtration rate	54.49	55.43	—
serum β2-microglobulin (mg/L)	**3.14**	**3.67**	1.3–3.0
Urine			
Protein	**2+**	Negative	Negative
Glucose	**3+**	**2+**	Negative
Total urine phosphorus in 24 h (mmol/24 h)	16.64	30.6	13–42
Urinary phosphorus concentration (mmol/L)	13.1	25.5	12.9–43.9

Notes: Bold values in the table indicate abnormal results.

## 3 Discussion

Fanconi syndrome is a rare renal tubular disorder caused by defects in the function transport of the proximal renal tubules, leading to renal tubular acidosis, hypophosphatemia, phosphaturia, renal glycosuria, aminoaciduria, hypokalemia, and hypocalcemia. The main clinical manifestations are bone pain, muscle weakness, growth retardation, polyuria, and polydipsia. Drug-induced nephrotoxicity is a common cause of acquired Fanconi syndrome, which may be associated with antiviral agents, chemotherapeutic agents, antibiotics and immunosuppressants. While acquired Fanconi syndrome can often be effectively treated by removing the causative agents, whether renal dysfunction caused by certain drugs can be fully reversible remains controversial (Koda et al., 2019; Qian et al., 2019; Viganò et al., 2011).

### 3.1 Pathogenesis of ADV-associated Fanconi syndrome

**FIGURE 3:**
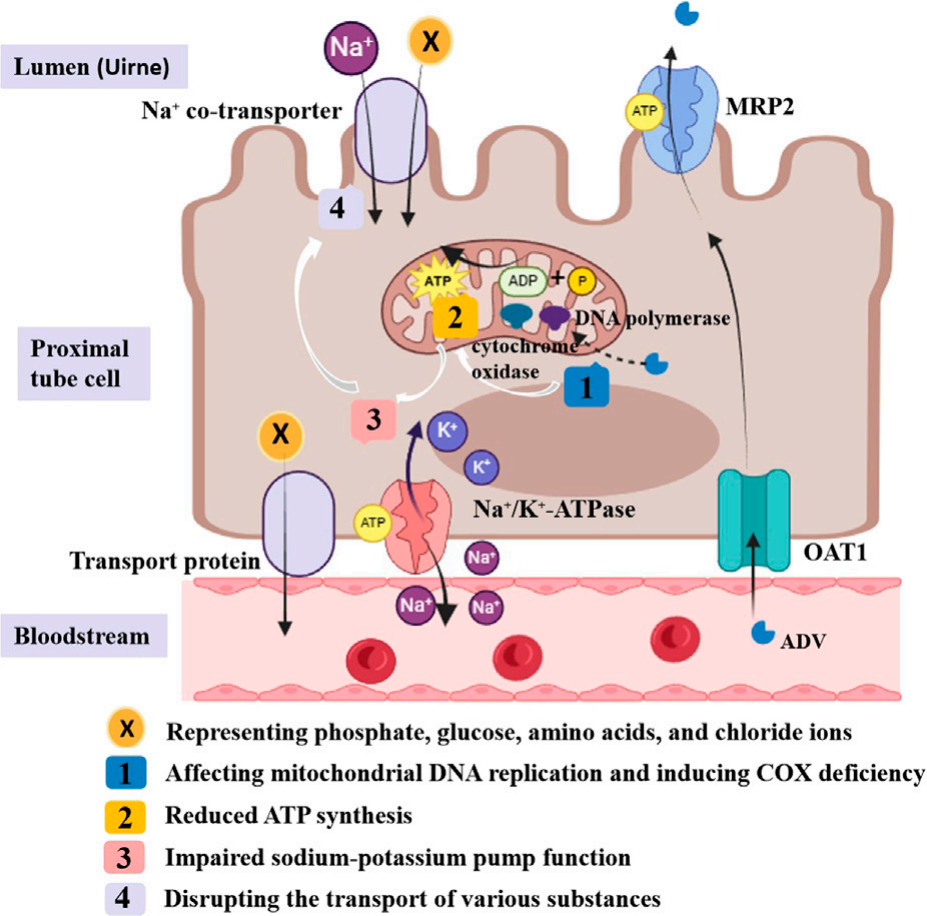
ADV excretion process on proximal tubule epithelial cells.

ADV can inhibit HBV-DNA replication by integrating phosphorylated adefovir diphosphate into viral DNA. It is primarily excreted via urine, absorbed by proximal tubules through basolateral organic anion transporter-1 (OAT1), and secreted into the lumen via apical multidrug resistance protein (MRP) (Izzedine et al., 2005; Cihlar et al., 1999; Shimizu et al., 2015). Long-term low-dose ADV use has been linked to Fanconi syndrome, potentially due to overexpression of OAT1 and inhibition of MRP (Izzedine et al., 2005; Tanji et al., 2001; Viganò et al., 2011). Additionally, its active metabolite, adefovir diphosphate, may impair energy metabolism by inhibiting mitochondrial DNA replication and inducing cytochrome oxidase deficiency. These effects cause ATP depletion, disrupting Na-K-ATPase pump function and the transport of various substances, including phosphate, glucose, and amino acids (Qi et al., 2015; Wu et al., 2013) (Figure 3). The precise pathophysiological mechanisms underlying hypophosphatemia-induced ATP dysregulation in Fanconi syndrome remain to be fully elucidated, positioning mitochondrial function as a promising investigative target for future research into this disorder.

### 3.2 Fanconi syndrome is often misdiagnosed as osteoporosis

Fanconi syndrome is often misdiagnosed as osteoporosis in clinical practice due to secondary hypophosphatemia impairing bone mineralization, leading to osteoporosis or osteomalacia. Hypophosphatemia, the most common abnormal laboratory finding in Fanconi syndrome, typically develops after 1 year of ADV treatment (Sun et al., 2020). Although our patient exhibited hypophosphatemia and osteoporosis during the consultation process but lacked typical osteomalacia features such as skeletal deformities or pseudofractures on imaging (Chao et al., 2022). In a literature review, the prevalence of glucosuria and proteinuria in patients with Fanconi syndrome was second only to hypophosphatemia (Sun et al., 2020). Therefore, electrolyte levels and urinalysis are important indicators for early diagnosis and disease monitoring.

There is some nephrotoxicity associated with long-term administration of ADV and the extent of renal damage is dose-dependent (Fontana, 2009). Although this patient had elevated creatinine and cystatin C levels in prior visits, the underlying cause was overlooked. Among renal function biomarkers, eGFR and cystatin C are widely recognized as more sensitive than serum creatinine, with eGFR often considered the optimal indicator (Lin et al., 2017). Additionally, urinary β2-M and retinol-binding protein (RBP) can help determine the optimal timing of antiviral regimen adjustments (Jia et al., 2015). A literature review emphasizes that patients with a pretreatment eGFR below 90 mL/min/1.73 m^2^ are at higher risk of renal injury (Lin et al., 2017). Therefore, clinicians, particularly hepatologists, should routinely assess renal function before and after treatment.

### 3.3 The association between Fanconi syndrome and peripheral neuropathy

Both hypophosphatemia attributable to Fanconi syndrome and peripheral neuropathy can cause muscle weakness, but there are distinct differences between the two conditions. Muscle weakness caused by peripheral neuropathies typically correlates with the distribution of affected nerves and may coexist with sensory deficits. Patient’s tendon reflexes are often reduced or absent. Conversely, hypophosphatemia-induced muscle weakness is usually generalized and may present with non-neuromuscular manifestations like bone pain and psychiatric disturbances (Han and Liu, 2024). It can be acutely exacerbated within a short period of time due to an increased demand for energy metabolism. But we cannot simply ascribe the cause of muscle weakness in this case to either isolated hypophosphatemia or peripheral nerve damage.

Surprisingly, after the replacement of antiviral drugs, phosphorus supplementation and neurotrophic treatment, our patient not only experienced resolution of muscle weakness and normalization of electrolyte levels but also showed improvement in EMG findings. Although there are several confounding factors such as the ADV cessation, the vitamin B supplemention, we speculate that there is a potential association between peripheral neuropathy and Fanconi syndrome.

To date, reports of adefovir dipivoxil-associated Fanconi syndrome complicated by peripheral neuropathy have been exceptionally rare (Lu et al., 2014), consistent with the scarce reports involving other nucleotide analogues such as tenofovir. Moreover, none of these studies compared the results of electromyography before and after the intervention. No mechanistic studies have explicitly stated the relationship between Fanconi syndrome itself, the hypophosphatemia and renal impairment it induces, and peripheral neuropathy. Michael’s study (Vanneste and Hage, 1986) reported a case of hypophosphatemia deriving from excessive parenteral nutrition, which presented with a clinical pattern similar to acute Guillain-Barré syndrome. The study hypothesized that the decreased utilization of glucose by red blood cells, along with reduced levels of 2,3-diphosphoglycerate and ATP, may lead to a leftward shift of the hemoglobin-oxygen dissociation curve, resulting in decreased tissue oxygen tension. Insufficient oxygen supply fails to meet the metabolic demands of cells, thereby contributing to the manifestation of neurological symptoms and signs. Schubert speculated from an experimental study in dogs that phosphate deficiency may alter the permeability characteristics of the muscle cell membrane by affecting ATP synthesis. This subsequently leads to changes in the transmembrane potential of skeletal muscles, ultimately resulting in muscle weakness (Fuller et al., 1976). However, a cross-sectional investigation by Yuan discovered that low phosphate showed no significant correlation with muscle strength and muscle mass (Chen Y. Y. et al., 2018). These findings suggest that impaired energy metabolism secondary to hypophosphatemia may contribute to the development of peripheral neuropathies. Further mechanistic studies are needed to validate this hypothesis in the future.

## 4 Conclusion

Patients on long-term ADV therapy should undergo regular monitoring of urine tests, electrolytes, and renal function, particularly in those with renal impairment. Hypophosphatemia caused by Fanconi syndrome may damage peripheral nerves by disrupting their energy metabolism. Further clinical and experimental evidence is required to clarify this mechanism.

## Data Availability

The original contributions presented in the study are included in the article/supplementary material, further inquiries can be directed to the corresponding authors.
